# The Relationship between Mitochondrial Respiratory Chain Activities in Muscle and Metabolites in Plasma and Urine: A Retrospective Study

**DOI:** 10.3390/jcm6030031

**Published:** 2017-03-10

**Authors:** Corinne Alban, Elena Fatale, Abed Joulani, Polina Ilin, Ann Saada

**Affiliations:** 1 Monique and Jacques Roboh Department of Genetic Research, Hadassah-Hebrew University Medical Center, P.O. Box 12000, 91120 Jerusalem, Israel; korin@hadassah.org.il; 2 Metabolic Laboratory, Department of Genetics and Metabolic Diseases, Hadassah-Hebrew University Medical Center, P.O. Box 12000, 91120 Jerusalem, Israel; fatalelena@yahoo.com (E.F.); abedg@hadassah.org.il (A.J.); ilin@hadassah.org.il (P.I.)

**Keywords:** mitochondrial disease, mitochondrial respiratory chain, plasma amino acids, plasma carnitines, urine organic acid, FGF21

## Abstract

The relationship between 114 cases with decreased enzymatic activities of mitochondrial respiratory chain (MRC) complexes I-V (C I-V) in muscle and metabolites in urine and plasma was retrospectively examined. Less than 35% disclosed abnormal plasma amino acids and acylcarnitines, with elevated alanine and low free carnitine or elevated C4-OH-carnitine as the most common findings, respectively. Abnormal urine organic acids (OA) were detected in 82% of all cases. In CI and CII defects, lactic acid (LA) in combination with other metabolites was the most common finding. 3-Methylglutaconic (3MGA) acid was more frequent in CIV and CV, while Tyrosine metabolites, mainly 4-hydroxyphenyllactate, were common in CI and IV defects. Ketones were present in all groups but more prominent in combined deficiencies. There was a significant strong correlation between elevated urinary LA and plasma lactate but none between urine Tyrosine metabolites and plasma Tyrosine or urinary LA and plasma Alanine. All except one of 14 cases showed elevated FGF21, but correlation with urine OA was weak. Although this study is limited, we conclude that urine organic acid test in combination with plasma FGF21 determination are valuable tools in the diagnosis of mitochondrial diseases.

## 1. Introduction

Mitochondria are essential organelles present in eukaryotic cells. They perform vital roles in many cellular pathways; however, their main function is to supply cellular energy in the form of adenosine triphosphate (ATP) via oxidative phosphorylation (OXPHOS) performed by the mitochondrial respiratory chain (MRC). The MRC located in mitochondrial inner membrane is comprised of ninety proteins organized into five multi-subunit enzymatic protein complexes. These proteins and many other auxiliary proteins are essential for maintaining MRC, and OXPHOS is encoded by two genomes, the nuclear genome and the mitochondrial genome (mtDNA). Consequences of OXPHOS dysfunction include not only energy (ATP) depletion but also elevated oxidative stress, disturbed mitochondrial membrane potential, subsequently leading to imbalanced calcium homeostasis, autophagy/mycophagy, apoptosis, and ultimately to cell death. Mitochondrial diseases affecting one or more MRC complexes are common (prevalence 5 to 15 cases per 100,000 individuals), disabling, progressive, or fatal disorders affecting several vital organs including the brain, optic nerves, the liver, skeletal muscles, and the heart, manifesting at birth, during infancy, and in adulthood [1,2,3]. They are mostly caused by mutations in genes leading to dysfunction of one or multiple MRCs or auxiliary proteins crucial for OXPHOS but could also be secondary to other conditions [4]. The extreme heterogeneity of mitochondrial disorders makes diagnosis complex and sometimes requires the investigation of MRC function in the affected tissues. Before an invasive procedure such as muscle biopsy is performed, a metabolic workup is usually performed in blood and/or urine. Exome sequencing has recently become a valuable diagnostic tool; however, the interpretation is complex when numerous pathogenic and/or new variants are detected [1,2,3,5]. In this retrospective study, we investigated the relationship between common metabolic tests and MRC dysfunction detected in muscle in order to facilitate the diagnostic workup of mitochondrial diseases.

## 2. Materials and Methods

### 2.1. Mitochondrial Respiratory Chain Enzymatic Analysis

The enzymatic activities of MRC complexes I-V and citrate synthase CS (a mitochondrial control enzyme) were determined by spectrophotometric methods in isolated muscle mitochondria, as we have previously described [6].

### 2.2. Metabolic Tests

Acylcarnitines were determined by electrospray–tandem mass spectrometry in plasma or dry bloodspots (Micromass, Waters, Milford, MA, USA) [7]. Organic acids in urine were determined qualitatively by gas chromatography–mass spectrometry (GC-MS) (Agilent, Santa Clara, CA, USA) [8]. Plasma amino acid analysis was performed on a Biochrom 30 amino acid analyzer according to the manufacturer’s instructions (Biochrom, Holliston, MA, USA).

### 2.3. Fibroblast Growth Factor 21

Fibroblast growth 21 (FGF 21) was determined in plasma, by a solid phase sandwich enzyme-linked immunosorbant assay using the Human FGF-21 Quantikine ELISA kit (R&D systems, Minneapolis, MN, USA) according to the manufacturer’s instructions.

### 2.4. Statistical Analysis

Where indicated, the relationship between two parameters was assessed by the Spearman’s rank-order correlation test using IBM SPSS statistics for Windows, version 24.0 (IBM Co., Armonk, NY, USA).

## 3. Results

### 3.1. Distribution of MRC Deficiencies

**Figure 1:**
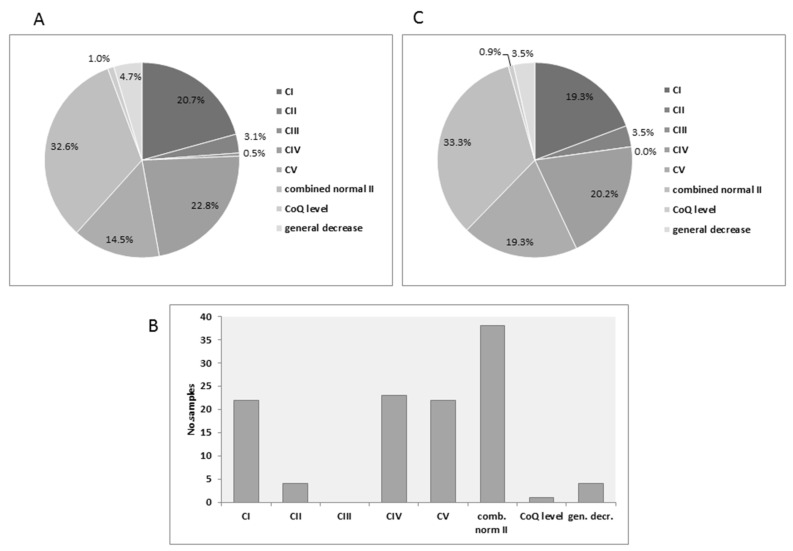
Distribution of MRC deficiencies: (**A**) The relative proportion of MRC defects according to groups. (**B**) The number of samples from each group with available metabolic test data. (**C**) The relative proportion of MRC defects with available metabolic test data.

Enzymatic analysis of MRC complexes I-IV was performed in 1163 muscle samples, referred to our laboratory for diagnostic purposes over a ten-year period (2006–2016). Mean age of the patients was 4.9 years ranging between 1 day and 67 years. Of these, 193 were found to be defective (17% diagnostic yield) in one or several MRC complexes (<50% residual activity of control mean, normalized to CS) (Figure 1A). The most common defect found (63 samples) were combined deficiencies including two or more MRC complexes but with normal or elevated CII activity. Other frequent defects were isolated CI (40 samples) and CIV (44 samples). Less frequent were deficiencies in CV (28 samples) and CII (6 samples). The rarest isolated defect was CIII with only one sample. Two cases which were designated “CoQ level” as the combined activity of CI + III and CII + III were decreased, while each measured separately disclosed normal activity. We also included a group designated as a general decrease where all respiratory chain complexes activities were decreased relative to CS. Metabolic workup data obtained from urine and/or plasma for diagnostic purposes was available for 114 of the 193 (59%) patients with decreased MRC activities in muscle (Figure 1B). This distribution reflected the total deficiencies (Figure 1C), with the exception of CIII, where no data was available and the proportion of CV defects somewhat increased. For comparison, we also obtained one or more metabolic parameters from 150 patients with clinical suspicion of mitochondrial diseases but with normal activities of MRC complexes I-V.

### 3.2. Plasma Amino Acids

**Figure 2:**
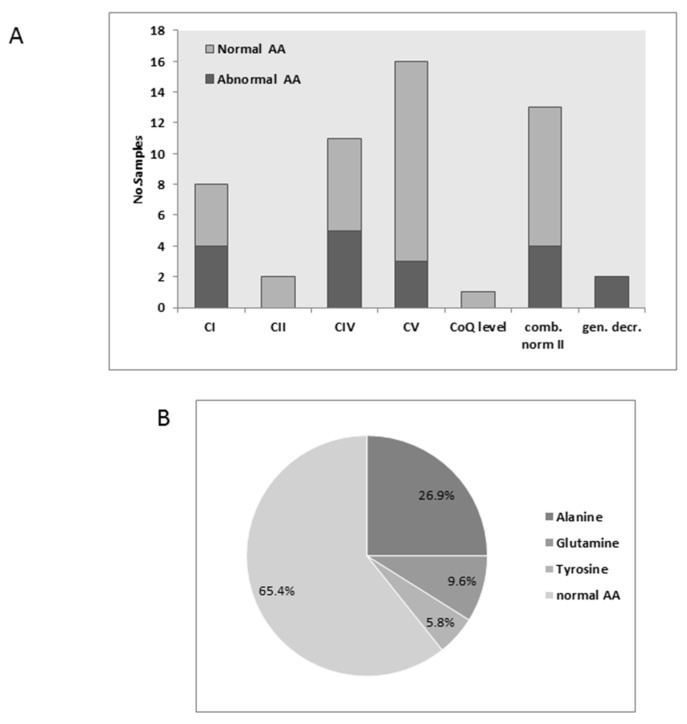
Plasma amino acids: (**A**) The relative proportion abnormal to abnormal plasma amino acid (AA) tests results according to groups; (**B**) The relative proportion of the most frequent abnormalities.

Plasma amino acids were measured in 49 samples but of these only 17 (34%) were abnormal, mostly disclosing elevated Alanine in alone or in combination with elevated Glutamine and/or tyrosine. Thus 50% or more in each group tested normal with the exception of two generally decreased cases that were both abnormal (Figure 2A). The most frequent abnormality was Alanine followed by Glutamine and Tyrosine (Figure 2B). Although quantitative data are available for many samples (analysis performed in our laboratory) we opted not to include these measurements as some samples were reported as abnormal without quantification (data reported from other sources). 10 tests disclosed elevated Alanine only, 4 with elevated Alanine and Glutamine and 4 elevated Alanine Glutamine and tyrosine. Other amino acids were inconsistent, for example Citrulline was elevated in two samples while decreased in one and branched chain amino acids, Proline, Phenylalanine and Methionine were elevated in in one case each (results not shown). As Alanine is also derived from lactate via transamination of pyruvate we assessed the correlation to plasma lactate in 19 samples with data available for both parameters; however, the association was weak at best and statistically not significant. Only 9 of 90 (10%) of cases with normal MRC disclosed abnormal plasma amino acids of these, 3 had elevated Alanine. Taken together, the amino acid analysis was not very informative with respect to predicting MRC dysfunction but is vital for the differential diagnosis of other conditions such as urea cycle disorders.

### 3.3. Acylcarnitines

**Figure 3:**
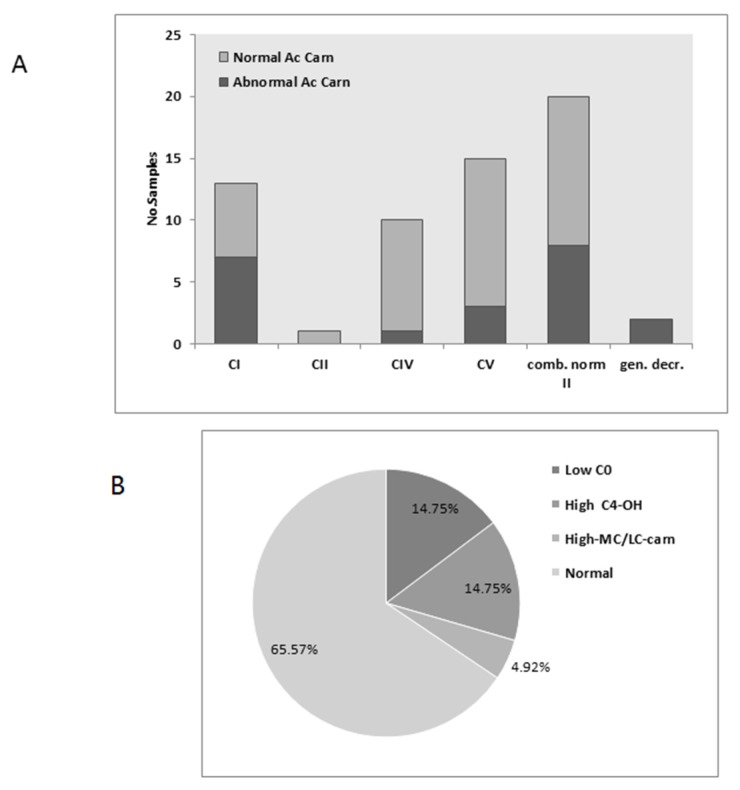
Plasma acylcarnitines: (**A**) The relative proportion abnormal to abnormal acylcarnitines (Ac. Carn) tests results according to groups; (**B**) The relative proportion of the most common abnormalities.

Acylcarnitine analysis test results were available from either plasma or dry bloodspots from 61 cases, of which 21 (34%) were abnormal. e most affected groups are CI defects, and approximately half were abnormal, as were the two cases of general defects. In the other groups, the test was mostly normal (Figure 3A). The most common abnormalities were low free C0-carnitine and elevated C4OH-(3-hyroxybutyryl)-carnitine. Elevation of medium- or long chain- (MC/LC) carnitines was only detected in two CV defects and one combined deficiency case (Figure 3B). Since elevated C4OH– carnitine is associated with ketosis, we evaluated the association with urinary ketones as detected by the organic acid analysis (see next section) from 17 samples and did not find any statistically significant correlation between the two parameters. Of 101 tests from patients with normal MRC, only nine (9%) had abnormal acylcarnitines, mostly with decreased in free carnitine and only one showing elevated C4OH-carnitine. Accordingly, as was the case with amino acid analysis, the acylcarnitine test was not significantly informative with respect to most MRC defects, but abnormality was more frequent than in cases with normal MRC.

### 3.4. Urinary Organic Acids

**Figure 4:**
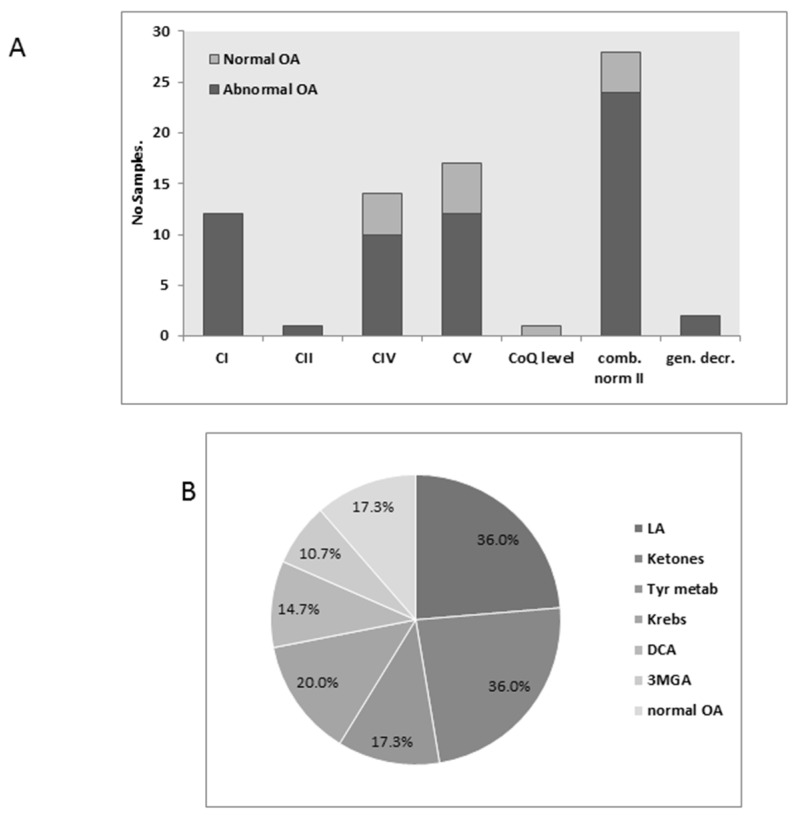
Urinary organic acids: (**A**) The relative proportion abnormal to abnormal urinary organic acids (OA) tests results according to groups; (**B**) The relative proportion of the most common abnormalities.

**Figure 5:**
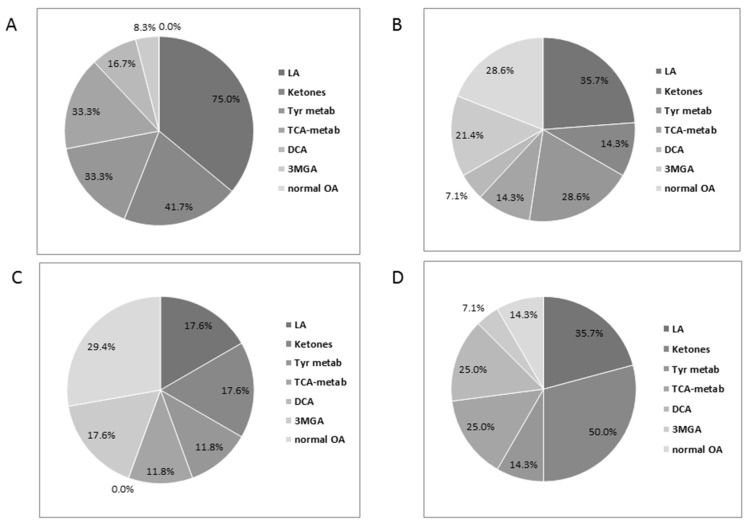
Urinary organic acids in (**A**) CI defects; (**B**) CIV defects; (**C**) CV defects; (**D**) combined defects with normal CII.

Urinary organic acids were qualitatively evaluated in 75 cases, of which 66 (82%) disclosed elevated levels of one or more metabolites. Notably, all samples in the groups CI and CII, and the general decrease, were abnormal, as was the majority of CIV and CV, and the combined defects. The single CoQ level sample tested normal (Figure 4A). The most frequent abnormalities were lactic acid (LA) concomitantly with ketones and/or TCA (Krebs cycle) metabolites and/or Tyrosine metabolites and/or dicarboxylic acids (DCA) and/or 3-methylglutaconic acid (3MGA). In all groups together, LA and ketones (mainly 3-hydroxybutyrate) were equally common followed by TCA and tyrosine metabolites (mainly 4-hydroxyphenyllactate), whereas 3MGA and DCA were less common (Figure 4B). Other occasionally occurring metabolites were methylmalonic acid, branched chain ketoacids, glutaric acid, and acylglycines but without any specific pattern (not shown). Interestingly, the distribution of the metabolites varied according the MRC defects (Figure 5).

In CI defects, 75% excreted elevated levels of LA, while this proportion was less than 36% or less in the other groups (Figure 5A). The combined defects group is characterized by a higher proportion of ketones (50%) and DCA (25%) (Figure 5D). Both CIV and CV defects disclosed a higher percentage of 3MGA than the other groups. However, almost a third of the samples in this group were normal (Figure 5B,D). Tyrosine metabolites (mainly 4-hydroxyphenyllactate) were common in groups CI and CIV. For comparison, 32 out of 88 (36%) cases with normal MRC disclosed an abnormal organic acid test; however, no specific pattern resembling the findings in MCR defects was recognized. For example, four cases had elevated ketones, six elevated LA, and four elevated Tyrosine metabolites, but mostly separately, LA was never observed in combination with another metabolite. On the contrary, in the MRC defective group, LA was elevated concomitantly with ketones and/or Tyrosine metabolites in 22 (91%) of 24 samples with elevated lactate. Interestingly, the “normal” group contained six samples with low/moderately elevation of methylmalonic acid in combination with other metabolites, while only one case was detected in the MRC defect group. This finding could possibly be related to a nutritional deficiency (vitamin B12) rather than an inborn metabolic disease. Consequently, the organic acid test is quite informative, and the results could be helpful in guiding the investigation towards a specific group of MRC defects. The number of cases with CII and III, and the general defects, was too small to be evaluated. Still, one out of two CII cases disclosed elevated succinate and fumarate. As expected, the correlation between urinary and plasma LA examined in 23 samples was statistically significant (R 0.843 P0.0). Additionally, we examined the relationship between Tyrosine metabolites in urine and plasma Tyrosine in 10 samples and did not find any statistically significant correlation, as only two samples disclosed elevated Tyrosine.

### 3.5. Plasma FGF21

The level of plasma FGF21 was measured in 14 samples, and all except one disclosed high levels (331–10,700 pg/mL (Normal < 250 pg/mL). Notably, eight samples with high FGF21 were found in the combined group with normal complex II. Nevertheless, we did not find any significant relationship to the urinary organic acid test. The correlation with urinary OA was at best weak (R 0.377, P0.356), as four samples with high FGF21 showed normal urinary OD, while one sample with normal FGF21 had an abnormal urinary organic acid profile. We also measured FGF 21 in 25 cases with normal MRC of which four disclosed elevated FGF21. Still, taken together, the correlation between abnormal MRC activities and plasma FGF21 is significant (R0.690, P0.0).

## 4. Discussion

The purpose of this study was to retrospectively examine the correlation between biochemical findings and MRC enzymatic activity in an unbiased manner. Obviously, this study is limited as it includes neither clinical (not reported in a systematic manner) nor molecular data (not available for most cases; however, from our limited knowledge, we estimate that at least 20% of the cases are molecularly defined). We made an effort, but were unable to establish correlations between the clinical symptoms and the different groups. We kindly refer to the literature for this complex issue [1,2,3,4,5]. Moreover, only data obtained from muscle are presented since we did not have pertinent data for liver tissue; therefore, conditions such as certain mtDNA depletion syndromes with variable tissue expression could have been overlooked in this study [9]. The limit of less than 50% residual activity normalized to citrate synthase was determined to potentially include cases of mtDNA heteroplasmy, and, from our experience, this cutoff is relevant [10]. On the other hand, a partial deficiency could also be secondary [4] to other conditions linked or apparently not linked to MRC function, as exemplified by decreased cytochrome c oxidase activity in Troyer syndrome [11], thus the inclusion criteria is indeed a complex, unresolved issue. Additionally, we did not include cases with pyruvate dehydrogenase E1 or E3 deficiency. Nevertheless, this study provides some pertinent information that could facilitate the decision of how to proceed with the workup of a patient clinically suspected to harbor a mitochondrial disease, and provides a complement to other studies [1]. According to the presented data, plasma amino acids did not disclose a significant correlation with MRC dysfunction as most showed a normal profile while a small proportion disclosed mainly elevated Alanine without significant correlation with plasma LA. We also did not detect any specific correlation with Citrulline and/or Arginine, as has been previously reported in MELAS [12], the reason for this, could be that our cohort did not include this specific condition. According to our data, acylcarnine test is also not very informative. Theoretically, impaired MRC should affect mitochondrial fatty acid oxidation with subsequent accumulation of acylcarnitine; however, we mostly detected signs of ketosis or decreased free carnitine. These findings are in accord with the consensus reported by the Mitochondrial Medicine Society [5]. Obviously, if indicated, both amino acid and acylcarnitine tests are important to rule out urea cycle disorders, amino acid degradation defects, fatty acid oxidation defects, primary/secondary carnitine deficiency, etc. and should therefore not be neglected. It is also anticipated that patients exhibiting abnormal acylcarnitine or amino acids, patterns consistent with a known inborn error of metabolism, would not be referred for a muscle biopsy. Still in the context of mitochondrial diseases urinary organic acid analysis seems to be considerably more informative, as the four out of five groups of MRC defects disclosed abnormally elevated excretion of one of several metabolites. Moreover, it was possibly to link certain patterns with certain MRC defects. LA and ketones, mostly concurrently, were prevalent in CI and combined defects. Notably, the combined defects encompass combinations of CI,III,IV,V defects, while complex II, which is solely encoded by the nuclear genome, is normal, so this group includes both mtDNA depletion and translation defects [1,9,13]. 3MGA aciduria is relatively prevalent in CIV and CV defect and is among several other disorders, been linked TMEM70 mutations in CV [14]. Recently elevated urinary 3MGA levels were reported in mitochondrial membrane defects with or without enzymatic MRC dysfunction, thus 3MGA remains an important biomarker for mitochondrial disease [15,16]. Although quantitative measurement in urine was not performed, elevated urine and LA were, as expected, closely correlated, serving as an additional marker of hyper lactic acidemia >6–10 mM [17]. Elevated 4-hydroxyphenyllactate, a Tyrosine metabolite in urine, and Tyrosine in plasma are also associated with liver dysfunction [18]. In this respect, the organic acid analysis is more sensitive as a marker since many cases showed increased 4-hydroxyphenyllactate while only a few had elevated Tyrosine. The more prevalent occurrence of urinary tyrosine metabolites in CI and CIV indicate liver involvement in the pathogenesis of these conditions. The significant correlation between abnormal MRC activities and plasma FGF21 confirms that this protein is a good marker for MRC dysfunction in accord with previously reported findings [19]. Moreover, the finding that elevated FGF21 was prevalent in the combined group and suspected of mitochondrial translation or maintenance defects is certainly consistent with the recent report by Lehtonen et al. [20]. Nevertheless, as some discrepancies were observed between plasma FGF21 and urine organic acids, it seems that the combination of these two tests would be more informative than each one alone. As we have previously pointed out, this study is limited, and there are several other biochemical parameters that were not included in this because of a lack of sufficient data. Among these are growth differentiation factor 15, amino acids, liver function tests, pyruvate, muscle pathology, etc. [5,20,21]. Nevertheless, according to our findings, testing patients suspected of a mitochondrial diseases, for organic acids in urine and FGF21 in plasma is informative and the results facilitate the decision whether to perform a biopsy or not. Alternatively, or in addition, these tests could also be useful in guiding the filtering of variants in exome analysis.

We conclude that urine organic acid test in combination with plasma FGF21 determination are valuable tools in the diagnosis of mitochondrial diseases.
